# Untangling the Influence of Heat Stress on Crop Phenology, Seed Set, Seed Weight, and Germination in Field Pea (*Pisum sativum* L.)

**DOI:** 10.3389/fpls.2021.635868

**Published:** 2021-03-29

**Authors:** Amrit Lamichaney, Ashok K. Parihar, Kali K. Hazra, Girish P. Dixit, Pradip K. Katiyar, Deepak Singh, Anil K. Singh, Nitin Kumar, Narendra P. Singh

**Affiliations:** ^1^ICAR-Indian Institute of Pulses Research, Kanpur, India; ^2^ICAR-Indian Agricultural Statistics Research Institute, New Delhi, India

**Keywords:** heat stress, seed germination, growing degree days, seed set, seed loss, GGE biplots

## Abstract

The apparent climatic extremes affect the growth and developmental process of cool-season grain legumes, especially the high-temperature stress. The present study aimed to investigate the impacts of high-temperature stress on crop phenology, seed set, and seed quality parameters, which are still uncertain in tropical environments. Therefore, a panel of 150 field pea genotypes, grouped as early (*n* = 88) and late (*n* = 62) maturing, were exposed to high-temperature environments following staggered sowing [normal sowing time or non-heat stress environment (NHSE); moderately late sowing (15 days after normal sowing) or heat stress environment-I (HSE-I); and very-late sowing (30 days after normal sowing) or HSE-II]. The average maximum temperature during flowering was about 22.5 ± 0.17°C for NHSE and increased to 25.9 ± 0.11°C and 30.6 ± 0.19°C in HSE-I and HSE-II, respectively. The average maximum temperature during the reproductive period (RP) (flowering to maturity) was in the order HSE-II (33.3 ± 0.03°C) > HSE-I (30.5 ± 0.10°C) > NHSE (27.3 ± 0.10°C). The high-temperature stress reduced the seed yield (24–60%) and seed germination (4–8%) with a prominent effect on long-duration genotypes. The maximum reduction in seed germination (>15%) was observed in HSE-II for genotypes with >115 days maturity duration, which was primarily attributed to higher ambient maximum temperature during the RP. Under HSEs, the reduction in the RP in early- and late-maturing genotypes was 13–23 and 18–33%, suggesting forced maturity for long-duration genotypes under late-sown conditions. The cumulative growing degree days at different crop stages had significant associations (*p* < 0.001) with seed germination in both early- and late-maturing genotypes; and the results further demonstrate that an extended vegetative period could enhance the 100-seed weight and seed germination. Reduction in seed set (7–14%) and 100-seed weight (6–16%) was observed under HSEs, particularly in HSE-II. The positive associations of 100-seed weight were observed with seed germination and germination rate in the late-maturing genotypes, whereas in early-maturing genotypes, a negative association was observed for 100-seed weight and germination rate. The GGE biplot analysis identified IPFD 11-5, Pant P-72, P-1544-1, and HUDP 11 as superior genotypes, as they possess an ability to produce more viable seeds under heat stress conditions. Such genotypes will be useful in developing field pea varieties for quality seed production under the high-temperature environments.

## Introduction

In the tropical climate, the evident adverse impacts of rising ambient temperature on cool-season crops are nowadays challenging production sustainability. The latest projections indicate that ambient temperature will rise by about 2–4°C by the end of the 2100 (IPCC, 2007). Almost all the cool-season legumes under the northern plains of India are gradually shifting toward “warm winter,” exposing crop to terminal heat stress (Basu et al., 2009). Terminal heat stress often causes a considerable yield loss in the late-sown field pea (Parihar et al., 2020b). To date, much is known about the agronomic, physiological, and molecular basis of crop responses to terminal heat stress. However, the possible impacts of heat stress on seed set, seed viability, and vigor of cool-season legumes in the tropical environments has not been studied adequately.

Field pea (*Pisum sativum* L.) is an imperative, highly productive, and nutritionally rich cool-season legume crop, grown across the world, consumed as food, feed, and fodder (Parihar et al., 2016, 2020a; Rubiales et al., 2019). India is a major field pea-producing country (FAOSTAT, 2019) and has a high demand for quality field pea seeds. Cool-season grain legumes including field pea have increased sensitivity to high-temperatures than warm-season grain legumes (Hall, 2001). Also, field pea has relatively low heat tolerance than other winter legumes like chickpea and lentil (Siddique, 1999), and so very often, the production declines when the maximum day temperature during flowering exceeds 25°C (Guilioni et al., 2003; Sadras et al., 2013). The impact of high-temperature on crop growth, physiology, and yields of field pea has been reported from different agro-regions (Sadras et al., 2013; Liu N. et al., 2019; Jiang et al., 2020; Mohapatra et al., 2020), but there are no reports on germination of seeds produced at high-temperature stress.

Quality seed production is quintessential to sustain national food and nutritional security. Seed quality, which is often determined by seed morphology, seed dormancy, germination, germination rate, and vigor, is predominantly influenced by the environmental conditions prevailing during the crop-growing season and subsequently during processing and storage (Peltonen-Sainio et al., 2011; Hampton et al., 2013; Rashid et al., 2018; Lamichaney et al., 2019). Among the climatic variables, the higher ambient temperature has an immense influence on growth and developmental processes of cool-season legumes and, thus, is anticipated to affect the seed quality traits. On account of the higher seed requirement of field pea, the possible adverse impact of high-temperature could have an enormous impact on seed growers. This is possible that the high-temperature stress would hamper seed germination and vigor by shortening the seed filling period that affects the transport and accumulation necessary to assimilate in the developing seed (Young et al., 2004; Shinohara et al., 2006). Assessment of high-temperature stress on crop phenology, seed set, and seed germination across a large set of genotypes would be a realistic approach to develop strategic management option(s) and to identify potential genotype(s) having improved and stable seed quality traits, in particular germination.

Development of high-yielding stable genotypes with quality seed production ability under heat stress environments (HSEs) is the need of the hour for achieving sustainable pulses production under the changing climates. Understanding of effects of genotypes (G) and genotype × environment (G × E) interaction is essential for selecting superior genotype(s) for the studies involving multi-environments (Yan et al., 2007), since seed yield and its quality are complicated traits strongly influenced by G × E interaction and, therefore, should not be judged on the basis of G and E means alone (Ebdon and Gauch, 2002). The delineation of G × E interactions requires multi-environmental testing of different genotypes for identification of superior genotype for the “trait of interest” based on mean performance and stability (Fan et al., 2007). During recent years, genotype main effect along with genotype × environment interaction (GGE) biplot is being used to depict G and G × E interactions graphically and also has dominance over additive and multiplicative models (Yan and Kang, 2003; Yan et al., 2007). Therefore, a comprehensive study was undertaken to elucidate the impact of high-temperature on crop phenology and harvested seed quality in a panel of 150 field pea genotypes for identification of superior genotype(s). The major hypotheses of the study were: (1) the high-temperature and temperature intensity would affect the field pea seed set and seed germination in tropical agro-region, (2) crop phenology and high-temperature intensity during flowering and reproductive period (RP) would have direct associations with seed production and seed quality traits under HSEs, and (3) there would be differential responses of field pea genotypes to high-temperature environments (G × E interaction) for seed production and seed viability. The findings of the study would be valuable for developing future crop management strategies for quality seed production and identification of superior field pea genotype(s) for the anticipated high-temperature era.

## Materials and Methods

### Site Characteristics

**FIGURE 1:**
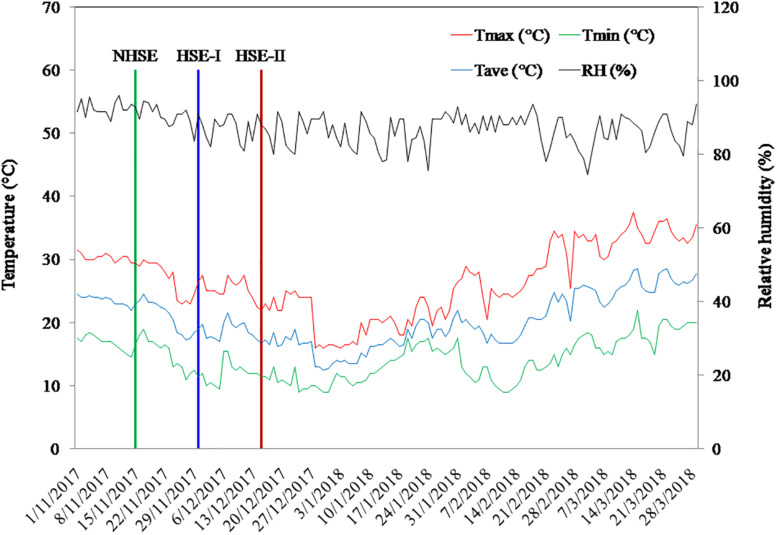
Average daily weather conditions of the experimental site during the period of study.

The field experiment was conducted during the winter season of 2017–2018 at ICAR-Indian Institute of Pulses Research, Kanpur, Uttar Pradesh, India (26°27′N latitude and 80°14′E longitude and approximately 152 m above mean sea level). The experimental soil is sandy-loam in texture and belongs to the order *Fluvisol* (World Reference Base classification). The climate of the site is sub-tropical humid with an average annual temperature of 26°C and annual rainfall of 720 mm. The soil of the experimental site (0–0.15 m) had pH 7.98, electrical conductivity 0.342 dS m^–1^, soil organic carbon 4.3 g kg^–1^, soil available nitrogen 102 mg kg^–1^, phosphorus 7.1 mg kg^–1^, and potassium 113 mg kg^–1^. The weather variables during the crop-growing season are presented in Figure 1.

### Planting Material and Experimental Details

A panel of 150 diverse genotypes of field pea including breeding lines, germplasm accessions, and high-yielding released cultivars were selected for the study (Supplementary Table 1). Selected genotypes based on maturity period were grouped as early- (≤110 days, *n* = 88) and late-maturing (>110 days, *n* = 62) genotypes. The selected genotypes were evaluated in three contrasting environments differing in the temperature intensity at crop stages (terminal heat stress in particular). This was achieved through sowing in three distinct windows [November 15 (normal sowing time), November 30 (moderately late sowing), and December 15 (very late sowing)] so as to expose field pea crop to HSEs. The HSEs simulated by delayed sowing were designated as heat stress environment-I (HSE-I) (moderately late sowing), heat stress environment-II (HSE-II) (very late sowing), and non-heat stress environment (normal sowing time) as non-HSE (NHSE). The experiment was laid out in augmented block design. In each environment, there were six blocks, and two checks [IPF 99-25 (early maturing) and IPF 5-19 (late maturing)] were replicated in each block.

### Crop Management

The experimental field was prepared by two ploughing followed by harrowing and planking. Three lines of each genotype were sown with a plant spacing of 30 cm × 5 cm. A filler spacing of 60 cm was kept between the genotypes. At the time of sowing, fertilizer nitrogen, phosphorus, and potassium was applied at 20–50–50 kg ha^–1^ (N–P_2_O_5_–K_2_O) as a basal dose. The irrigation was scheduled based on irrigation water/cumulative pan evaporation (IW/CPE) ratio of 0.75. For NHSE, the irrigation was given at 40 and 78 days after sowing (DAS), where irrigation was scheduled at 30 and 67 DAS for HSE-I and 51 and 73 DAS for HSE-II. For all the genotypes, one hand-weeding at 25 DAS was done to control the seasonal weeds.

### Observations on Phenology

Crop phenophases were determined by visual observations. For each genotype, the days from sowing to 50% flowering (when flowering appeared in 50% of the total plants) were denoted as days to 50% flowering (DTF). Likewise, the maturity date of each genotype was recorded when all the pods turned to light-yellow color and fully dried, and days from sowing to maturity was denoted as days to maturity (DTM). The difference between DTF and DTM was designated as RP and expressed as days.

### Seed Set

At maturity, five plants of each genotype were randomly selected, and 10 pods from the top 4 pod-bearing nodes were collected for observation on seed set percentage. The number of fully developed seeds per pod (>5-mm diameter) and the number of ovules per pod were counted and computed to derive seed set percent for each genotype across the three environments. A total of 4,500 pods were examined to compute the percent seed set across the three environments (3 environments × 150 genotypes × 10 pods).

$$\mathrm{Seed}\mathrm{set}\left(\%\right)=\frac{\mathrm{Number}\,\mathrm{of}\,\mathrm{fully}\,\mathrm{developed}\,\mathrm{seeds}\,\mathrm{per}\,\mathrm{pod}}{\mathrm{Number}\,\mathrm{of}\,\mathrm{ovules}\,\mathrm{per}\,\mathrm{pod}}$$

### Seed Yield and 100-Seed Weight

The inner row (4-m length) was harvested separately to estimate the final seed yield. The harvested seeds were sun-dried, and moisture percentage was calculated. The seed yield was adjusted at 12% moisture content and expressed as kg ha^–1^. The 100 seeds of each genotype were manually counted and weighed for determination of 100-seed weight. The 100-seed weight was calculated in three replications.

### Seed Germination Test

One hundred pure seeds in three replications were placed inside moist germination paper and then incubated at 20°C in dark for 8 days. On the ninth day of incubation, the seedlings and seeds were grouped into normal and abnormal seedlings, hard and dead seeds, respectively (ISTA, 2016). The percentage of normal seedlings to total seeds represents the final germination percentage.

Germination rate was calculated according to Maguire (1962). Briefly, 25 seeds of each genotype in three replications were placed in Petri plates lined with two pre-wetted filter paper and were incubated at 20°C in dark for 8 days. The number of seeds germinated was counted daily. The seeds were considered germinated when radicle attained an approximate length of 2 mm.

$$\text{Germination}\,\text{rate}=\,\sum_{n=1}^{8}\frac{\mathrm{Number}\,\mathrm{of}\,\mathrm{seedlings}\,\mathrm{germinated}\,\mathrm{in}\,n^{\text{th}}\,\text{day}}{n}$$

### Seed Loss Calculation

Total seed loss in stressed environments (HSE-I and HSE-II) was calculated based on the seed yield and germination loss as compared with the non-stressed environment (NHSE) with the following formula:

$$\mathrm{Seed}\,\mathrm{loss}\,\mathrm{in}\,\mathrm{HSE}-\text{I}\,\left(\mathrm{kg}\,{\mathrm{ha}}^{-1}\right)=[\mathrm{Seed}\mathrm{yield}\mathrm{in}\mathrm{NSHE}\left(\mathrm{kg}{\mathrm{ha}}^{-1}\right)\times$$

$$\text{germination}\left(\%\right)\mathrm{in}\mathrm{NHSE}-SeedyieldinHSE-I\left(\mathrm{kg}{\mathrm{ha}}^{-1}\right)\times$$

germination(%)inHSE-I]/100

$$\mathrm{Seed}\,\mathrm{loss}\,\mathrm{in}\,\mathrm{HSE}-\text{II}\,\left(\mathrm{kg}\,{\mathrm{ha}}^{-1}\right)=$$

$$[\mathrm{Seed}\mathrm{yield}\mathrm{in}\mathrm{NSHE}\left(\mathrm{kg}{\mathrm{ha}}^{-1}\right)\times$$

$$\text{germination}\left(\%\right)\mathrm{in}\mathrm{NHSE}-\mathrm{Seed}\mathrm{yield}\mathrm{in}\mathrm{HSE}-\text{II}\left(\mathrm{kg}{\mathrm{ha}}^{-1}\right)\times$$

germination(%)inHSE-II]/100

### Temperature Intensity and Growing Degree-Day Calculation

The average ambient maximum temperature (°C) during flowering (i.e., at 50% flowering ± 5 days) was calculated for each genotype to determine the temperature intensity during the flowering stage. Likewise, the cumulative GDD were calculated for vegetative (sowing to flowering), reproductive (flowering to maturity), and full crop seasons (sowing to maturity) of each field pea genotype and denoted as GDD_VEG_, GDD_RP_, and GDD_FS_, respectively. The GDD value at different crop growth stages was calculated using the following formula:

Growing⁢degree⁢day(C∘)=∑(TMAX+TMIN2-base⁢temperature)

where *T*_MAX_ and *T*_MIN_ are the maximum and minimum temperatures, and the base temperature is defined as the minimum threshold temperature below which the crop development is stopped. The base temperature in field pea is 0°C.

### Statistical Analyses

The data of each genotype were adjusted following the augmented block design analysis. The percent germination data were subjected to arcsine transformation before statistical analysis. The multivariate regression analysis was performed using “Data Analysis Toolpak” Add-Ins of Microsoft Excel. The principal component analysis (PCA) was done in PAST 3.14. Genotype + genotype-by-environment (GGE) biplots were developed using R studio platform using the “GGEBiplotGUI” package (Yan and Tinker, 2006). The GGE biplots were constructed by plotting the first two principal components derived by subjecting mean values to singular value decomposition (Parihar et al., 2017a,b). To display the mean performance and stability of a genotypes, the biplots were framed with the mean vs. stability function by adopting no scaling (scale = 0) and tester-centered G + GE (centering = 2) with genotype focused (row metric preserving) singular value partitioning (SVP = 1). Conversely, the efficiency of environments was portrayed by discriminativeness vs. representativeness function with no scaling (scale = 0) and tester-centered G + GE (centering = 2) with symmetrical (genotype–environment focused) singular value partitioning (Parihar et al., 2018).

## Results

### High-Temperature Stress

**FIGURE 2:**
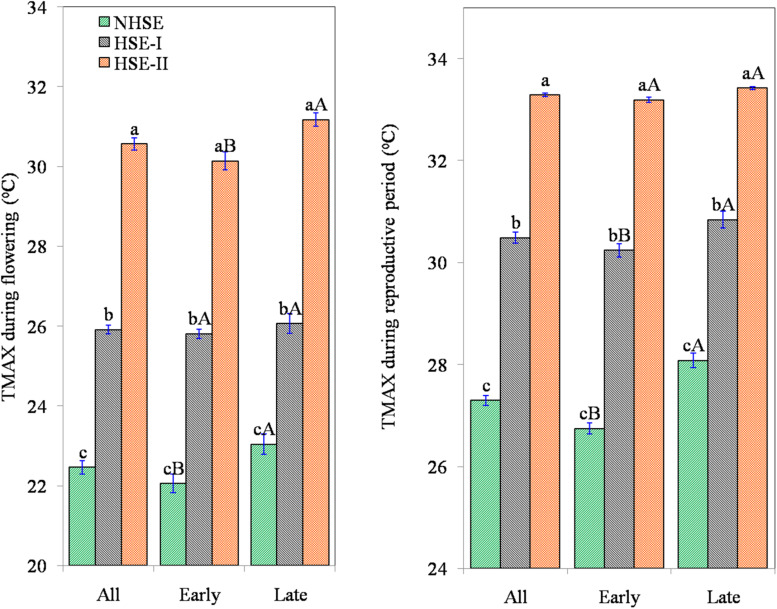
Exposure of field pea genotypes to maximum temperature(*T*_MAX_) during 50% flowering (°C) and average ambient temperature during reproductive period (°C) under timely sown and late-sown conditions. The error bars represent ± standard error of means. NHSE, normal sowing time; HSE-I, 15 days after NHSE; HSE-II, 30 days after NHSE. Different lowercase letters (a–c) represent significant different within the row values (*p* < 0.05). Different uppercase letters (A, B) represent significant difference within the column values (early and late genotypes) (*p* < 0.05).

Delayed sowing exposed the crop to considerable extent of heat stress especially during post-flowering stages (Figure 2). At flowering, the crop faced a maximum temperature (*T*_MAX_) of 22.5 ± 0.17°C in NHSE, while in HSE-I and HSE-II, the *T*_MAX_ was 25.9 ± 0.11°C and 30.6 ± 0.15°C, respectively. Likewise, the average *T*_MAX_ during the RP was 27.3 ± 0.10°C, 30.5 ± 0.10°C, and 33.3 ± 0.03°C for NHSE, HSE-I, and HSE-II, respectively. The reduction in crop season GDD was prominent (22–26%, *p* < 0.01) in HSE-I and HSE-II over NHSE (Table 1). The GDD_VEG_ (sowing to flowering) and GDD_RP_ (flowering to maturity) were reduced in the late-sown crops, and the reduction was much prominent in the late-maturing over early-maturing genotypes. In HSE-II, a strong reduction in GDD values was observed in both vegetative (26%) and reproductive (23%) stages as compared with NHSE (*p* < 0.05).

**TABLE 1:** Crop stage-wise growing degree days (°C) under different growing environments.

Period		Notation	Genotype group	Sowing time		
			
From	To			NHSE	HSE-I	HSE-II
Sowing	Harvest	GDD_FS_	Early	1,982.91.5^aB^	1,953.29.9^bB^	1,530.87.3^cA^
			Late	2,142.214.3^aA^	2,009.211.3^bA^	1,541.16.7^cA^
			All	2,048.78.8^a^	1,976.37.7^b^	1,535.15.0^c^
Sowing	Flowering	GDD_VEG_	Early	1,253.815.4^aB^	1,248.710.7^aB^	931.87.9^bB^
			Late	1,326.918.8^aA^	1,279.215.3^aA^	966.77.2^bA^
			All	1,284.011.2^a^	1,261.38.1^a^	946.25.6^b^
Flowering	Harvest	GDD_RP_	Early	729.115.2^aB^	704.511.9^aB^	599.08.5^bA^
			Late	815.322.8^aA^	730.017.1^bA^	574.49.8^cB^
			All	764.711.8^a^	715.08.9^b^	588.96.3^c^

*Different lowercase letters (a–c) represent significant difference (*p* < 0.05) within the row values (between environments). Different uppercase letters (A, B) represent significant difference (*p* < 0.05) within the column values (between early and late genotypes). Value represents mean ± standard error of mean.*

### Phenological Events

**FIGURE 3:**
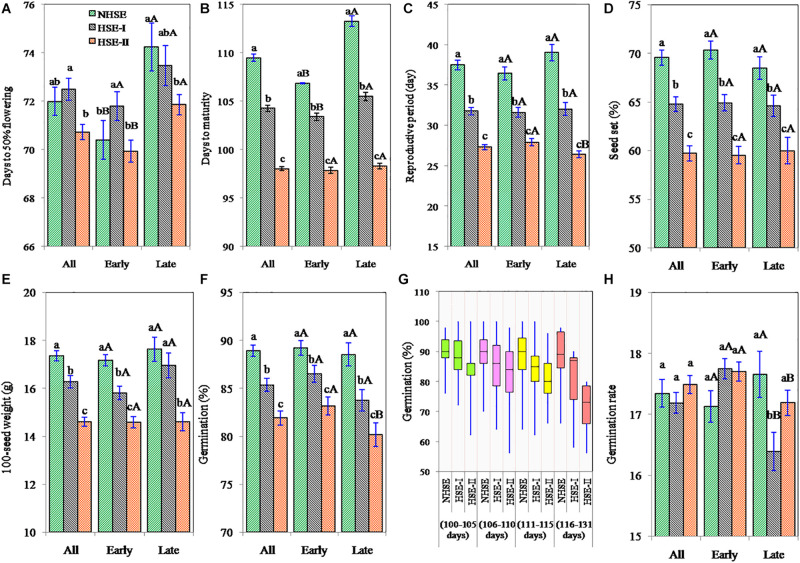
Days to 50% flowering **(A)**, days to maturity **(B)**, reproductive period **(C)**, percent seed set **(D)**, 100-seed weight **(E)**, seed germination **(F,G)**, and germination rate **(H)** of early, late, and overall field pea genotypes under timely sown and late-sown conditions. The error bars represent mean ± standard error of means. Different lowercase letters (a–c) represent significant difference (*p* < 0.05) within the row values (between environments). Different uppercase letters **(A,B)** represent significant difference (*p* < 0.05) within the column values (between early and late genotypes).

Results showed that the crop-growing environments had marginal influence on length of the vegetative period (Figure 3A), while under late-sown conditions (HSE-I and HSE-II), the RP and DTM were reduced by 15–27 and 5–11%, respectively, higher for late-maturing genotypes (6–13 and 18–32%, respectively) (Figures 3B,C). The average maturity period of early-maturing genotypes was 103 and 98 days in HSE-I and HSE-II, respectively. Likewise, for late-maturing genotypes, the average maturity duration in HSE-I and HSE-II was 106 and 98 days, respectively. The reduction in RP under HSE-I and HSE-II for early-maturing genotypes was 12 and 22%, respectively, and for late-maturing genotypes, the corresponding reductions were 16 and 30%. The average RP in NHSE was 37.5 days, which reduced to 31.8 days in HSE-I and further to 27.3 days in HSE-II.

### Seed Set and Seed Weight

High-temperature stress affected seed setting in field pea (Figure 3D). The reduction in seed set in HSE-I and HSE-II was 7 and 15% in early-maturing genotypes (*p* < 0.05), 6 and 12%, respectively, in late-maturing genotypes (*p* < 0.05). Results further showed that in tropical environment, seed setting in field pea had a wider genotypic variation (38–100%), and an average 33% of the ovules failed to set seeds.

The reduction in 100-seed weight in early-maturing genotypes was 8% (*p* < 0.05) and 15% (*p* < 0.05) under HSE-I and HSE-II over NHSE (Figure 3E), respectively. Likewise, the reduction in the late-maturing genotypes was 4% (*p* > 0.05) and 17% (*p* < 0.05) under HSE-I and HSE-II environments, respectively. We observed higher 100-seed weights with the decrease in the seed set values in all the environments; however, a significant negative (*r* = −0.20, *n* = 150) association was observed in HSE-I only. A significant negative association (*p* < 0.001) was found at 100-seed weight with *T*_MAX_ at flowering and *T*_MAX_ during the RP, while 100-seed weight showed strong positive associations with GDD_VEG_, GDD_RP_, and GDD_FS_ in both early- and late-maturing genotypes (Table 2).

**TABLE 2:** Pearson correlation coefficient (*r*) between selected parameters.

Genotype	TMAX_F_	TMAX_F–M_	GDD_VEG_	GDD_RP_	GDD_FS_	HSW	HSW

	HSW	HSW	HSW	HSW	HSW	Germination	Germination rate
Early (*n* = 264)	−0.414***	−0.420***	0.260***	0.278***	0.362***	−0.008	−0.149*
Late (*n* = 186)	−0.364***	−0.427***	0.348***	0.240***	0.387***	0.167*	0.148*
All (*n* = 450)	−0.394***	−0.423***	0.291***	0.252***	0.361***	0.073	−0.016

*Significant at p < 0.05, ***Significant at p < 0.001.

### Seed Germination

The seeds harvested from NHSE, HSE-I, and HSE-II environments had the average seed germination of 89, 85, and 82%, respectively, showing the average reduction of 4 and 8% at HSE-I and HSE-II as compared with NHSE (Figure 3F). The maximum reduction (>15%) in seed germination was recorded in long-duration genotypes (>115 days) harvested from HSE-II, although the detrimental changes in seed germination under HSEs were highly genotype-specific and both early- and late-maturing genotypes were found in the higher germination loss group in HSE-I [>20% loss, *n* = 8 (4E + 4L)] and HSE-II [>20% loss, *n* = 16 (7E + 9L)]. Meanwhile, the stable genotypes (for germination) were dominated by genotypes < 114 days of maturity group. Genotypes IPF 5-19 (125 days) and IPFD 16-3 (107 days) were found to be highly unstable genotypes for seed germination (>25% seed germination loss) under heat stress condition. The reduction in germination of long-duration genotypes (>115 days) was 16% in HSE-II, while the reduction in short-duration genotypes (<105 days) was only 4% (Figure 3G). However, no consistent changes were observed in germination rate of seeds harvested from normal and HSEs (Figure 3H).

### Genotypic Variation, Trait Association, and Multivariate Analysis

Results revealed that ample amount of variability existed for all the studied traits in different environments, i.e., HSE-I, HSE-II, and NHSE (Supplementary Table 2). Correlation results showed a negative associations (*p* < 0.001) between seed germination and *T*_MAX_ during flowering (*r* = −0.301) and RP (*r* = −0.268) (Table 3), whereas the multivariate regression analysis revealed that *T*_MAX_ during RP had a prominent negative impact on seed germination over *T*_MAX_ during flowering for both the early- and late-maturing genotypes. The growing degree days for vegetative period (GDD_VEG_) had a positive association with seed germination (*p* < 0.001) (Table 4).

**TABLE 3:** Regression equations and association between germination and seed set in field pea with ambient maximum temperature.

Parameter (*Y*)	Independent variables (*X*)	Genotype group	*n*	Intercept (*a*)	Slope (*b*)	*t*-stat	*r*	*p* value
Germination (%)	*T*_MAX_ during flowering (°C)	Early	264	100.1	−0.53	−3.98	−0.239	<0.001
		Late	186	102.6	−0.69	−4.05	−0.286	<0.001
		All	450	101.7	−0.062	−5.89	−0.301	<0.001
Germination (%)	Average *T*_MAX_ during RP (°C)	Early	264	111.3	−0.83	−4.74	−0.281	<0.001
		Late	186	119.9	−1.162	−4.28	−0.301	<0.001
		All	450	115.2	−0.98	−5.89	−0.268	<0.001
Seed set (%)	*T*_MAX_ during flowering (°C)	Early	264	93.02	−1.08	−6.58	−0.377	<0.001
		Late	186	82.3	−0.67	−3.57	−0.255	<0.001
		All	450	88.6	−0.091	−7.37	−0.329	<0.001
Seed set (%)	Average *T*_MAX_ during RP (°C)	Early	264	106.7	−1.39	−6.33	−0.364	<0.001
		Late	186	98.3	−1.11	−3.74	−0.266	<0.001
		All	450	103.6	−1.28	−7.38	−0.329	<0.001

**Parameter (Y)**	**Genotype group**	***n***	**Regression equation**	***R*^2^**	**Adj. *R*^2^**	***p* value**			
	
						**Regression**	**Intercept**	**TM_F_**	**TM_RP_**

Germination	Early	264	*Y* = 115.9 + 0.403TM_F_ - 1.33TM_RP_	0.083	0.076	<0.001	<0.001	0.271	0.006
	Late	186	*Y* = 119.7 - 0.013TM_F_ - 1.143TM_RP_	0.094	0.084	<0.001	<0.001	0.977	0.120
	All	450	*Y* = 117.8 + 0.191TM_F_ - 1.232TM_RP_	0.091	0.087	<0.001	<0.001	0.498	0.002

**T*_MAX_, maximum temperature (°C); RP, reproductive period; TM_F_, *T*_MAX_ during flowering; TM_RP_, average *T*_MAX_ during reproductive period.*

**TABLE 4:** Regression equations and association between germination with growing degree days (GDD) in field pea.

Parameter (Y)	Genotypes group	*n*	Regression equation	*R*^2^	Adj. *R*^2^	*p* value			
	
						Regression	Intercept	GDD_VEG_	GDD_RP_
Germination	Early	264	*Y* = 72.05 + 0.0117GDD_VEG_ + 0.0012 GDD_RP_	0.065	0.058	<0.001	<0.001	<0.001	0.769
	Late	186	*Y* = 63.24 + 0.0164GDD_VEG_ + 0.0020GDD_RP_	0.127	0.117	<0.001	<0.001	<0.001	0.619
	All	450	*Y* = 71.65 + 0.0129GDD_VEG_ + 0.0010GDD_RP_	0.079	0.075	<0.001	<0.001	<0.001	0.731

**FIGURE 4:**
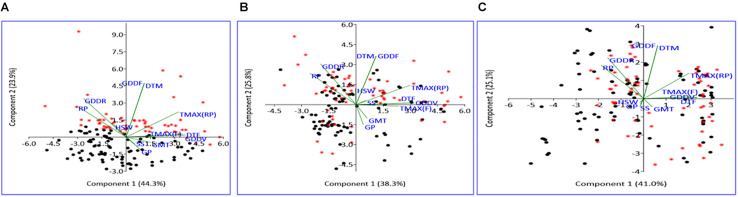
Scatter plot of field pea genotypes on PCA coordinates under NHSE **(A)**, HSE-I **(B)**, and HSE-II **(C)** environments. Black dots represent early-maturing genotypes, while red asterisk represents late-maturing genotypes. RP, reproductive period; GDDR, growing degree days at reproductive period; GDDF, growing degree days of full crop season; DTM, days to maturity; TMAX(RP), maximum temperature at reproductive period; HSW, 100-seed weight; TMAX(F), maximum temperature at flowering; DTF, days to 50% flowering; GDDV, growing degree days at vegetative period; GMT, germination rate; SS, seed set; GP, germination percent; PCA, principal component analysis.

The PCA conducted using weather and seed parameters revealed that early- and late-maturing genotypes response were distinct in NHSE, as these genotypes were clearly distinguished in different PCA coordinates. However, in heat stress environments (HSE-I and HSE-II), specific differential responses between early- and late-maturing genotypes were not observed, as they were found to be distributed all over the PCA coordinates. The loading coefficient values revealed the maximum contribution of variation by RP, DTF, TMAX_RP_, and TMAX_F_ for PC1, while DTM, GDD_RP_, and GDD_FS_ contributed the maximum for PC2 (Supplementary Figure 1). The negative association of germination with RP, DTM, and GDD_RP_, particularly under the HSEs observed by correlation studies, was further confirmed in PCA graph (Figure 4).

### Seed Loss

The total seed loss, which defined the combined loss of seed yield and seed viability, in early-maturing genotype was 309 and 737 kg ha^–1^ under HSE-I and HSE-II over NHSE, respectively. Likewise, for the late-maturing genotypes, the reduction was 361 and 835 kg ha^–1^ under HSE-I and HSE-II environments, respectively (Table 5).

**TABLE 5:** Seed loss (kg ha^–1^) in early- and late-maturing field pea genotypes under heat stress conditions.

Genotype group	Seed loss (kg ha^–1^)	
	
	HSE-I	HSE-II
Early	308.62 ± 45.90^bA^	736.72 ± 46.83^aA^
Late	361.08 ± 66.63^bA^	835.03 ± 54.21^aA^
All	330.30 ± 39.08^b^	777.36 ± 33.13^a^

*Different lowercase letters (a,b) represent significant difference (*p* < 0.05) within the row values (between environments). Similar uppercase letter (A) represent non significant difference (p > 0.05) within the column values (between early and late genotypes). Value represents mean ± standard error of mean.*

### Identification of Superior Genotypes and Environments for Germination Using GGE Biplots

**FIGURE 5:**
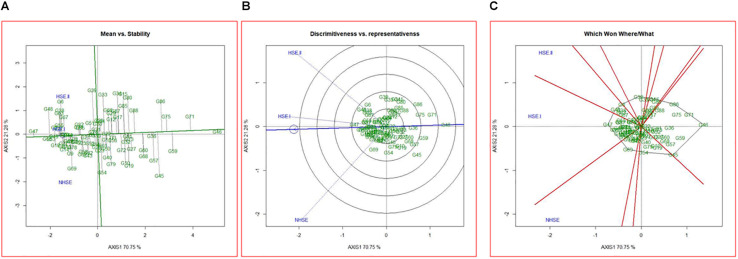
GGE biplots developed by plotting the first and second principal components derived from the germination value of early-maturing genotypes of field pea evaluated in three environments (NHSE, HSE-I, and HSE-II). **(A)** “Mean versus Stability” view of genotypes. **(B)** Discriminating ability and representativeness of three environments, **(C)** which won where/what of field pea genotypes under different environments.

**FIGURE 6:**
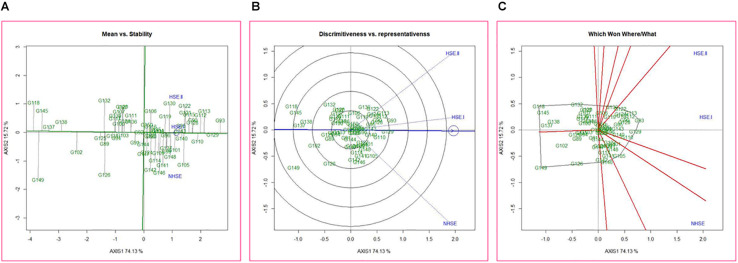
GGE biplots developed by plotting the first and second principal components derived from the germination value of late-maturing genotypes of field pea evaluated in three environments (NHSE, HSE-I, and HSE-II). **(A)** “Mean versus Stability” view of genotypes. **(B)** Discriminating ability and representativeness of three environments, **(C)** which won where/what of field pea genotypes under different environments.

**FIGURE 7:**
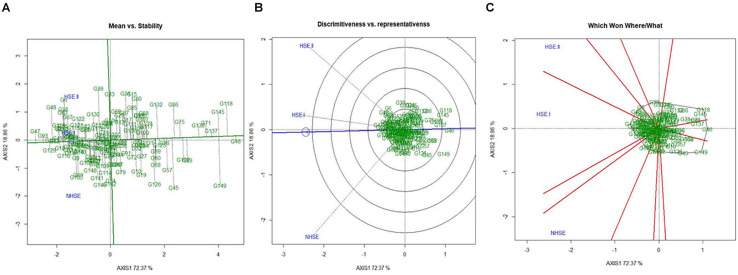
GGE biplots developed by plotting the first and second principal components derived from the germination value of all the genotypes of field pea evaluated in three environments (NHSE, HSE-I, and HSE-II). **(A)** “Mean versus Stability” view of genotypes. **(B)** Discriminating ability and representativeness of three environments, **(C)** which won where/what of field pea genotypes under different environments.

Mean performance and stability of the genotypes for germination efficiency across the environments, i.e., NHSE, HSE-I and HSE-II, were graphically represented through “average environment axis” (AEA) view of the biplot for early, late, and overall genotypes (Figure 5). These biplots explained 92, 90, and 91% of total variation in early, late, and overall genotypes, respectively, of the genotypes centered G × E. The single arrow line (AEA) in the graph passing through biplot origin indicates higher mean performance of the genotypes. Thus, among early-maturing genotypes, Pant P-72 (G47) had the highest germination value followed by P-1544-1 (G16) and IPFD 1-9 (G53). On the contrary, genotype IPFD 16-4 (G46) followed by EC 499761 (G71) had the lowest germination value (Figure 5A). Among the late-maturing genotypes, the best-performing genotypes were IPFD 11-5 (G93) and HUDP 11 (G129) for germination, while genotypes P-1041 (G118) and IPF 5-19 (G149) had the lowest germination (Figure 6A). Overall, Pant P-72 (G47) and IPFD 11-5 (G93) had the highest mean performance for germination percentage across the environments, while IPF 5-19 (G149) and P-1041 (G118) had the lowest germination percentage (Figure 7A). Genotypic stability is generally assessed on the basis of the absolute length of the projection of a genotype onto the AEA ordinate. The ideal genotypes would be those that have high mean performance and high stability (projection on AEA ordinate close to zero). Accordingly, IPFD 11-5 (G93) and P-1544-1 (G16) were the most superior genotypes with high mean performance and high stability for germination in all the environments. Genotypes situated nearer to the superior genotype are more “desirable” than others. The environment vector view of the GGE biplot based on germination efficiency of different category of genotypes illustrated the discriminating ability and representativeness of environments. In early category, HSE-II environment had highest discriminating ability followed by HSE-I. Further, the environment HSE-I was the highest representative (smallest angle with the AEA) followed by NHSE and HSE-II. Environments had positive correlation among each other, but HSE-II had high correlation with HSE-I and poor positive correlation with NHSE. Similarly, NHSE had high correlation with HSE-I and a weak positive association with HSE-II (Figure 5B). In case of late-maturing genotypes and overall genotypes, the environment HSE-II and HSE-I had high positive correlation (Figures 6B, 7B).

The biplot also indicates the most responsive genotypes (situated on the vertices of the polygon) at some or all environments. The performance of genotypes can also be evaluated based on GGE biplots. The genotypes IPF 227 (G49), Pant P-72 (G47), and HFP 9907B (G69) showed high germination value in HSE-II, HSE-I, and NHSE, respectively (Figure 5C). In the case of the late group, HSE-II and NHSE environments had the highest discriminating ability, while HSE-I was the most representative environment followed by HSE-II. The best-performing genotypes were IPF 17-19 (G113), IPFD 11-5 (G93), and IPF 99-31 (G105) in HSE-II, HSE-I, and NHSE, respectively, for germination (Figure 6C). In all sets of genotypes among the three environments, HSE-I had the highest representativeness along with high discriminating ability (Figure 7C).

## Discussion

The temperature data validate that the late-sown-induced targeted terminal HSEs were adequately attained in this study. The heat intensity (*T*_MAX_) during the flowering and RP varied largely among the environments, and the impact of HSEs was evident on most of the studied parameters. Late-sown-induced terminal HSE is widely used for field level screening of a large number of genotypes for heat-sensitive traits (Krishnamurthy et al., 2011; Huang et al., 2017; Jiang et al., 2020). Altered crop phenology under the stressful environments has been suggested as a crop adaptation strategy (Rezaei et al., 2015). In chickpea, G × E studies demonstrate the critical role of phenology in adaptation to high- and low-yielding eco-regions of India (Berger et al., 2006). Our results demonstrated that the impact of HSEs is more prominent on the RP than the vegetative period, and in all the genotypes, forced maturity occurred. However, the shortening of vegetative period in the long-duration genotypes under late-sown condition (HSE-II) was possibly attributed to the early exposure of crop to extreme heat events. The variations in the phonological events within the environments were directly reflected in the cumulative growing degree days for different crop stages.

Results demonstrate that the high-temperature stress reduces seed setting in the tropical environments. Seed set percentage was reduced by 7 and 14% when plants were exposed to the higher maximum temperatures of about 30.1 and 31.2°C during flowering. This finding contradicts the previous reports that seed set in pea was not affected at a higher temperature of up to 32°C (Jeuffroy et al., 1990; Guilioni, 1997; Jiang et al., 2015). Such contrasting results could be because of the differential heat sensitivity of the field genotypes, as a wider genotypic variation for seed set (38–100%) was observed in this study. Source–sink relations are often strongly influenced by the climatic variables, and a limiting source capacity also influences sink formation as a feedback mechanism that has been observed in other crops. The reduction in the number of seeds under elevated temperature may be an inherent protective mechanism adapted by the plant to produce fewer seeds. Equally, the reduction in the pollen formation and its viability causes lower seed setting at high-temperature in groundnut (Prasad et al., 1999; Kakani et al., 2002), cowpea (Ahmed et al., 1992), and chickpea (Devasirvatham et al., 2012). Jiang et al. (2015) have further reported a significant reduction in seed set of field pea at high-temperature stress of 36°C, attributed to reduced pollen germination and subsequent pollen tube growth. Loss in pollen viability, pollen number, pollen germination, pollen tube growth, shrunken pollen, empty pollen grains, failure in fertilization, and embryo abortion are some of the abnormalities associated with high-temperature stress in legumes (Liu Y. et al., 2019).

Our study showed that seeds produced at HSE-I and HSE-II had a lower seed weight than seeds produced at NHSE, demonstrating that an increased temperature during crop growth period in general and seed developmental stage in particular decreased seed weight. This relationship is further confirmed by significant negative correlations of 100-seed weight with *T*_MAX_ at flowering and RP. Similar findings have been reported in pea (Jiang et al., 2020) and other legumes such as *Vicia sativa* (Li et al., 2017) and French bean (Thomas et al., 2009). Reduction in the seed weight due to high-temperature stress during seed development may be attributed to forced maturity leading to faster rate of seed development and reduced seed filling period (Rolston et al., 1997; Young et al., 2004), which was likewise evident in the study. The crop average RP in NHSE was about 37 days, whereas HSE-II had the RP of 27 days. Such reduction in seed filling period due to high-temperature limits the transfer of assimilates and its accumulation in seed, resulting in reduced seed weight (Prasad et al., 2015). Besides this, high-temperature stress-induced reduction in seed weight could be attributed to reduction in the activities of enzymes involved in starch accumulation such as sucrose synthase and soluble and granule-bound starch synthase (Zhao et al., 2008). The elevated temperature during reproductive stages impedes the photosynthetic rate, synthesis of assimilates, and their translocation to flower, causing reduced pollen viability, stigma receptivity, and improper fertilization, which result in less and smaller-size grains (Farooq et al., 2017). Jeuffroy et al. (1990) also reported that the maximum temperature above 25°C during flowering or seed filling period impacted pea yield. The field pea crop when exposed to high-temperature (>25°C) at the end of its crop cycle leads to seed abortion, seed weight, and yield losses (Guilioni et al., 2003; Benezit et al., 2017; Parihar et al., 2020b).

The study showed that the terminal high-temperature stress not only affects the seed set or seed yield but also reduces the viability of the harvested seeds. The reduction in seed germination due to heat stress was more prominent in long-duration genotypes over short-duration genotypes. For instance, the reduction in germination of extra-long-duration genotypes (>115 days; *n* = 6) was 16% in HSE-II, while the reduction in extra short-duration genotypes (<105 days; *n* = 34) was only 4% (Figure 3G). The reduction in RP from 37 (NHSE) to 27 days (HSE-II) might have resulted into development of seeds with not enough reserve metabolites accumulated for normal development of seedling. High-temperature stress faced by the late-maturing genotypes in HSE-II led to a forced maturity with intense shrinkage (10 days) of grain filling period. Such genotypic differences in seed germination loss due to high-temperature suggest the possibility of screening and developing varieties of field pea that are suitable for pretended high-temperature era in future. Rashid et al. (2018) reported a negative effect of exposing *Brassica* plants to high-temperature on seed germination and attributed to production of increased proportion of abnormal seedlings upon high-temperature stress. Controlled production of reactive oxygen species (ROS) and adequate availability and supply of energy are important processes required for normal seed germination (McDonald, 1999). High-temperature led oxidative stress, which might have resulted in uncontrolled production of ROS, which could reduce the metabolic activity of the seed necessary for normal germination (Rashid et al., 2020). Such alteration in physiological and biochemical processes might have reduced the rate of seed germination in late-maturing genotypes at HSE-I.

Correlation and multivariate analysis demonstrated that high-temperature stress strongly influenced the seed weight. Also, the reduction in seed weight under late-sown HSEs influences the seed germination only for long-duration genotypes. Results further suggest that *T*_MAX_ during the RP had a higher adverse impact on seed germination over the *T*_MAX_ during flowering. The strong positive association between GDD_VEG_ and seed germination implies that extended vegetative period and pre-flowering reserve accumulation could result in improved seed quality in field pea under tropical climates.

The PCA results indicate maximum variability contributed by the weather parameters like RP, DTF, TMAX_RP_, and TMAX_F_ for PC1 and DTM, GDD_RP_, and GDD_FS_ for PC2. The GGE biplot analysis for germination efficiently explains the variability, as the first two principal components demonstrated around 90% of total variation in all groups of genotypes, i.e., early, late, and all, while GE had almost more than 15% share in total variation. The mean performance and stability analysis for germination demonstrated that genotypes, namely, IPFD 11-5 (G93), Pant P-72 (G47), P-1544-1 (G16), and HUDP 11 (G129), are the potential genotypes across the environments. In addition, these genotypes had very high PC1 values (high germination) and low PC2 values (high stability), in agreement with biplot analysis (Yan et al., 2007). Therefore, these genotypes could be valuable in field pea breeding programs. Conversely, genotypes that exhibit analogous germination pattern across environments were positioned closely on GGE biplot. Genotypes identified in present investigation could be helpful for breeders to develop pea varieties with high-quality seed production efficiency. The GGE biplot technique has been used to identify the best genotypes for trait of interest in many crops, i.e., yellow mosaic disease in mung bean (Parihar et al., 2017b), wilt and rust in lentil (Parihar et al., 2017a, 2018), rust in field pea (Das et al., 2019), and nematodes in mung bean (Singh et al., 2020). These findings indicated that genotypic response for germination was independent in all the environments. Therefore, unique genotypes should be chosen, and distinct selection strategies may be utilized for different environments. Notably, in HSE-II and HSE-I environments, the late-maturing genotypes demonstrated an analogous germination pattern. The correlation study showed a positive association (<90 angle) among all environments with varied magnitude. Furthermore, based on the discriminating ability and representativeness, HSE-I (moderate late planting) appeared to be an ideal testing condition for germination efficiency of field pea. For late genotypes out of two environments (HSE-II and HSE-I), anything can be dropped without losing much information about the genotypes; consequently, testing cost can be minimized and efficiency enhanced by using a minimum set of test environments. The high G × E interaction demonstrated in GGE biplot analysis suggests the existence of potential genotypic variation for seed quality parameter, which needs to be explored for crop improvement.

## Conclusion

The study concluded that heat stress in field pea affected crop phenology, seed set, and seed quality, higher in long-duration genotypes. However, substantial variation in seed quality traits was noticed within maturity group due to high-temperature stress. PCAs demonstrated a distribution pattern of diverse genotypes and traits across the environments. Furthermore, GGE biplot analysis for germination identified the promising genotypes, namely, IPFD 11-5 (G93), Pant P-72 (G47), P-1544-1 (G16), and HUDP 11 (G129), suitable for high-temperature stress and normal conditions. These genotypes would be a valuable source to speed up the breeding program for developing genotypes with high-quality seed production ability under heat stress conditions. Moreover, it is suggested that in order to meet the higher demand of quality seed, breeding for high yielding genotypes with quality seed production efficiency under high-temperature stress needs to be embraced to sustain pulses production under anticipated high-temperature stress environments.

## Data Availability Statement

The original contributions presented in the study are included in the article/Supplementary Material, further inquiries can be directed to the corresponding authors.

## Author Contributions

AP, AL, GD, and NS conceptualized the overall idea. AL, AP, and KH drafted the manuscript. DS, KH, and PK analyzed the data. AL, AS, and NK performed the phenotyping. GD, PK, and NS edited and finalized the manuscript. All authors contributed to the article and approved the submitted version.

## Conflict of Interest

The authors declare that the research was conducted in the absence of any commercial or financial relationships that could be construed as a potential conflict of interest.
