# Supplementary material for: Untangling the Influence of Heat Stress on Crop Phenology, Seed Set, Seed Weight, and Germination in Field Pea (Pisum sativum L.)
Source: Front Plant Sci. 2021 Mar 29;12:635868. doi: 10.3389/fpls.2021.635868 (PMC8040956; doi:10.3389/fpls.2021.635868)
Supplement: Supplementary file 1 [file Data_Sheet_1.docx]

**Supplementary Table 1. Detail list of genotypes along with their biplot code**

| **Genotype Biplot id** | **Genotype name** | **Genotype Biplot id** | **Genotype name** | **Genotype Biplot id** | **Genotype name** |
| --- | --- | --- | --- | --- | --- |
| G1 | P-91-3 | G26 | IPFD 12-8 | G51 | Pant P-25 |
| G2 | IPFD 98-1 | G27 | IPFD 14-2 | G52 | IPFD 98-9 |
| G3 | IPFD 10-12 | G28 | IPFD 15-5 | G53 | IPFD 1-9 |
| G4 | IPF 15-21 | G29 | IPF 16-18 | G54 | IPFD 2-5 |
| G5 | DDR 13 | G30 | P-1615 | G55 | EC 538004 |
| G6 | KPMR 385 | G31 | HUDP-8 | G56 | IPF 1-17 |
| G7 | DPFPD 2 | G32 | LMR-240 | G57 | IPF 3-13 |
| G8 | DPFPD 13 | G33 | KPMR 443 | G58 | IPF 3-17 |
| G9 | DPFPD 20 | G34 | Pant P-42 | G59 | IPF 6-16 |
| G10 | IPF 27 | G35 | IPFD 99-14 | G60 | IPF 6-20 |
| G11 | IPFD 14-11 | G36 | P-995 | G61 | IPF 7-16 |
| G12 | IPF 15-13 | G37 | KPMR-516 | G62 | IPFD 9-3 |
| G13 | IPF 16-13 | G38 | IPFD 98-7 | G63 | IPFD 10-13 |
| G14 | IPFD 17-2 | G39 | IPFD 99-13 | G64 | IPF 10-16 |
| G15 | IPFD 17-6 | G40 | IPFD 1-10 | G65 | IPF 12-20 |
| G16 | P-1544-1 | G41 | IPFD 2-6 | G66 | IPFD 13-2 |
| G17 | KPMR-602 | G42 | IPF 12-17 | G67 | IPF 13-13 |
| G18 | Pant P-31 | G43 | IPF 13-14 | G68 | Kushiamatar |
| G19 | AZAD P-4 | G44 | IPFD 15-8 | G69 | HFP 9907B |
| G20 | KPM 11-1 | G45 | IPFD 16-3 | G70 | IM 9102 |
| G21 | Pant P 101 | G46 | IPFD 16-4 | G71 | EC 499761 |
| G22 | DPFPD 62 | G47 | Pant P-72 | G72 | Azad P-1 |
| G23 | IPF 14 | G48 | EC 342002 | G73 | VL 46 |
| G24 | IPFD 98-4 | G49 | IPF 227 | G74 | LFP 224 |
| G25 | IPFD 12-2 | G50 | DDR 42 | G75 | VL-1 |

Cont….

Cont..

| **Genotype Biplot id** | **Genotype name** | **Genotype Biplot id** | **Genotype name** | **Genotype Biplot id** | **Genotype name** |
| --- | --- | --- | --- | --- | --- |
| G76 | IPFD 99-15 | G101 | HUDP 12 | G126 | IPF 8-20 |
| G77 | IPF 99-25 | G102 | EC 15056 | G127 | IPF 17-18 |
| G78 | IPFD 4-6 | G103 | ET 5120 | G128 | P-1429 |
| G79 | IPF 7-20 | G104 | ET 45189 | G129 | HUDP 11 |
| G80 | IPFD 11-10 | G105 | IPF 99-31 | G130 | P-1375 |
| G81 | IPFD 13-4 | G106 | IPF 2-13 | G131 | P-489 |
| G82 | KPMR 400 | G107 | IPF 2-19 | G132 | FC 1 |
| G83 | HUDP-7 | G108 | IPFD 3-7 | G133 | IM9101 |
| G84 | EC 595959 | G109 | IPFD 5-3 | G134 | LMR 20 |
| G85 | VPMR-9 | G110 | IPFD 8-1 | G135 | DMR-34 |
| G86 | EC 392177 | G111 | IPFD 8-14 | G136 | DMR 37 |
| G87 | AZAD P-2 | G112 | IPF 11-13 | G137 | PUSA 10 |
| G88 | VL 45 | G113 | IPF 17-19 | G138 | P-999 |
| G89 | IPF 99-26 | G114 | P-117 | G139 | P-471 |
| G90 | IPF 1-22 | G115 | TRCP-8 | G140 | IPFD 4-15 |
| G91 | IPFD 3-6 | G116 | EC 567770 | G141 | IPF 9-11 |
| G92 | IPF 10-21 | G117 | B-22 | G142 | IPF 9-17 |
| G93 | IPFD 11-5 | G118 | P-1041 | G143 | HUP-2 |
| G94 | IPF 14-16 | G119 | HFP9426 | G144 | IPFD 5-8 |
| G95 | IPF 14-13 | G120 | DMR 7 | G145 | P-1038 |
| G96 | ET-5117 | G121 | P-1657 | G146 | IPFD 6-3 |
| G97 | KFP 103 | G122 | LFP 212 | G147 | IPF 4-9 |
| G98 | KPMR-389 | G123 | IPF 4-26 | G148 | IPFD 9-2 |
| G99 | P-725 | G124 | IPF 5-23 | G149 | IPF 5-19 |
| G100 | JP 885 | G125 | IPFD 8-3 | G150 | IPF 11-5 |

**Supplementary Table 2**. Descriptive statistics of studied parameters for a panel of 150 diverse field pea genotypes under normal and heat stress environments

| Parameter | Environment | Min | Max | Mean | SE | CI (95%) |
| --- | --- | --- | --- | --- | --- | --- |
| Germination (%) | NHSE | 64 | 100 | 88.94 | 0.595 | 1.177 |
|  | HSE-I | 58 | 100 | 85.39 | 0.676 | 1.335 |
|  | HSE-II | 56 | 100 | 81.95 | 0.734 | 1.45 |
| Germination rate | NHSE | 5.5 | 22.83 | 17.35 | 0.229 | 0.453 |
|  | HSE-I | 8.0 | 21.33 | 17.19 | 0.172 | 0.34 |
|  | HSE-II | 10.52 | 22.62 | 17.49 | 0.15 | 0.296 |
| Days to flowering | NHSE | 56 | 91 | 71.99 | 0.587 | 1.159 |
|  | HSE-I | 62 | 86 | 72.49 | 0.447 | 0.884 |
|  | HSE-II | 63 | 78 | 70.73 | 0.314 | 0.62 |
| Reproductive period (days) | NHSE | 21 | 67 | 37.49 | 0.576 | 1.138 |
|  | HSE-I | 18 | 50 | 31.76 | 0.437 | 0.863 |
|  | HSE-II | 18 | 36 | 27.29 | 0.324 | 0.641 |
| Days to maturity | NHSE | 102 | 131 | 109.5 | 0.354 | 0.699 |
|  | HSE-I | 95 | 113 | 104.2 | 0.297 | 0.587 |
|  | HSE-II | 92 | 105 | 98.02 | 0.208 | 0.412 |
| Seed set (%) | NHSE | 38.02 | 99.64 | 69.21 | 0.703 | 1.389 |
|  | HSE-I | 45.79 | 93.39 | 64.8 | 0.771 | 1.523 |
|  | HSE-II | 37.85 | 82.69 | 59.73 | 0.777 | 1.536 |
| HSW (g) | NHSE | 10.11 | 32.26 | 17.37 | 0.219 | 0.434 |
|  | HSE-I | 8.294 | 29.68 | 16.29 | 0.252 | 0.498 |
|  | HSE-II | 8.316 | 25.32 | 14.61 | 0.193 | 0.382 |
| GDD_V_ (ºC-day) | NHSE | 999.1 | 1637 | 1284 | 11.18 | 22.1 |
|  | HSE-I | 1062 | 1526 | 1261 | 8.113 | 16.03 |
|  | HSE-II | 801.1 | 1073 | 946.2 | 5.551 | 10.97 |
| GDD_RP_ (ºC-day) | NHSE | 450.8 | 1473 | 764.7 | 11.77 | 23.26 |
|  | HSE-I | 421.3 | 1125 | 715 | 8.931 | 17.65 |
|  | HSE-II | 415.8 | 777.5 | 588.9 | 6.293 | 12.43 |
| GDD_FCS_ (ºC- day) | NHSE | 1867 | 2605 | 2049 | 8.846 | 17.48 |
|  | HSE-I | 1746 | 2209 | 1976 | 7.747 | 15.31 |
|  | HSE-II | 1386 | 1713 | 1535 | 5.003 | 9.886 |
| Tmax at flowering (ºC) | NHSE | 18.77 | 26 | 22.46 | 0.165 | 0.325 |
|  | HSE-I | 24.45 | 30.86 | 25.91 | 0.107 | 0.212 |
|  | HSE-II | 25.86 | 32.77 | 30.56 | 0.152 | 0.301 |
| Tmax during RP (ºC) | NHSE | 24.32 | 30.85 | 27.3 | 0.099 | 0.195 |
|  | HSE-I | 28.57 | 33.29 | 30.49 | 0.104 | 0.205 |
|  | HSE-II | 31.98 | 33.81 | 33.29 | 0.033 | 0.065 |
| Seed yield (kg ha^-1^) | NHSE | 363.5 | 2600.0 | 1238.4 | 34.56 | 68.30 |
|  | HSE-I | 170.54 | 1816.14 | 944.24 | 31.50 | 62.23 |
|  | HSE-II | 97.30 | 1252.76 | 496.67 | 17.81 | 35.20 |

Supplementary Table 3

ANOVA table of studied parameters

| Environment | Source of Variation | *df* | DTF | DTM | RP | SS | HSW | SY | GR | GP# |
| --- | --- | --- | --- | --- | --- | --- | --- | --- | --- | --- |
| NSHE | Block (ignoring treatments) | 5 | 932.6 | 1996.2* | 255.5** | 258.6 | 71.1** | 1158769** | 53.8* | 98.27* |
|  | Treatments (eliminating blocks) | 149 | 7441.5** | 2041.4** | 7697.8** | 11328.3** | 1021.0** | 29180764** | 1395.8** | 7717.6** |
|  | Checks | 1 | 816.8** | 675.0** | 12.0** | 610.1** | 11.2** | 1173688** | 241.6** | 463.2** |
|  | Genotypes + Checks vs Genotypes | 148 | 6624.7* | 1366.4** | 7685.8** | 10718.2** | 1009.8** | 28007075** | 1154.2** | 7254.4** |
|  | Error | 5 | 15.0** | 1.2** | 2.0** | 31.4* | 4.0** | 502.2** | 0.4** | 9.68** |
|  | Total | 159 | 8389.0 | 4038.9 | 7955.3 | 11618.4 | 1096.1 | 30340034.9 | 1450.0 | 7825.6 |
| HSE-I | Block (ignoring treatments) | 5 | 239.2 | 257.761* | 113.2 | 78.7 | 129.5** | 1235824** | 81.8* | 392.9 |
|  | Treatments (eliminating blocks) | 149 | 4797.8** | 1,887.76* | 4914.0** | 13682.7* | 1294.3** | 23609563** | 737.6** | 9607.3** |
|  | Checks | 1 | 675.0** | 128 | 768.0** | 113.6 | 5.0* | 6987** | 147.0** | 2092.2** |
|  | Genotypes + Checks vs Genotypes | 148 | 4122.8** | 1,887.77* | 4146.0** | 13569.1 | 1289.3** | 23602577** | 590.6** | 7515.1** |
|  | Error | 5 | 4.0* | 16.103** | 0.9** | 111.3* | 9.8** | 5.4** | 0.2** | 8.87** |
|  | Total | 159 | 5041.0 | 2289.6 | 5028.1 | 13872.6 | 1433.7 | 24845392.0 | 819.6 | 10009. 1** |
| HSE-II | Block (ignoring treatments) | 5 | 273.8 | 22.7** | 168.6 | 97.7 | 10.9** | 244106** | 17.0** | 313.8* |
|  | Treatments (eliminating blocks) | 149 | 2048.6** | 1048.2** | 2394.0** | 14109.2** | 838.1** | 6940889** | 534.3** | 9010.8** |
|  | Checks | 1 | 75.0** | 48.0** | 243.0** | 759.8** | 3.9** | 108120** | 41.8** | 1366.1** |
|  | Genotypes + Checks vs Genotypes | 148 | 1973.6** | 1000.2** | 2151.0** | 13349.4** | 834.2** | 6832770** | 492.5** | 7644.7** |
|  | Error | 5 | 4.1** | 1.1** | 0.6** | 28.5 | 2.6** | 46.0** | 2.1** | 2.77** |
|  | Total | 159 | 2326.6 | 1072.0 | 2563.2 | 14235.4 | 851.5 | 7185040.9** | 553.3 | 9327.4 |

*significance at P < 0.05; ** significance at P < 0.01.

#arcsine transformed data of GP was used for ANOVA

DTF, days to 50% flowering; DTM, days to maturity; RP, Reproductive period; SS, seed set; HSW, 100-seed weight; SY, seed yield; GR, Germination rate; GP, germination percent

**Loading coefficient value**

Supplementary figure 1: Loading coefficient value according to PCA analysis for different environments
